# Renal Cell Carcinoma in End-Stage Renal Disease: A Review and Update

**DOI:** 10.3390/biomedicines10030657

**Published:** 2022-03-11

**Authors:** Ziad M. El-Zaatari, Luan D. Truong

**Affiliations:** 1 Department of Pathology and Genomic Medicine, Houston Methodist Hospital, Main Building, Houston, TX 77030, USA; ltruong@houstonmethodist.org; 2 Weil Medical College, Cornell University, New York, NY 10022, USA

**Keywords:** end-stage renal disease, renal cell carcinoma, acquired cystic kidney disease, clear-cell papillary renal cell carcinoma, pathology, molecular pathology, immunohistochemistry, end-stage renal disease-associated renal cell carcinoma, acquired cystic kidney disease-associated renal cell carcinoma

## Abstract

Renal cell carcinoma (RCC) occurring in the setting of end-stage renal disease (ESRD) shows unique clinicopathological characteristics. The two most frequent types of ESRD-associated RCC are acquired cystic kidney disease-associated renal cell carcinoma (ACKD-RCC) and clear-cell papillary renal cell carcinoma (ccpRCC). Other types of RCC also occur in ESRD, albeit with different frequencies from the non-ESRD general population. The histological features of RCC do not vary in the setting of ESRD vs. non-ESRD, yet other findings, such as multifocality and multiple tumor types, are more frequent in ESRD. Studies have generated novel and important knowledge of the etiology, epidemiology, diagnosis, treatment, immunophenotype, and molecular characteristics of ESRD-associated RCC. Knowledge of these data is important for both pathologists and other physicians who may encounter ESRD patients with RCC. This review presents a comprehensive summary and update of the literature on RCC in ESRD, with a focus on the two most frequent types, ACKD-RCC and ccpRCC.

## 1. Introduction

The prevalence of end-stage renal disease (ESRD) requiring renal replacement therapy is estimated at 4–7 million cases worldwide [1] and the prevalence of ESRD continues to rise [2]. It has been recognized that patients with ESRD have an increased propensity for developing renal cell carcinoma (RCC) at rates higher than patients without ESRD [3,4,5]. Acquired cystic kidney disease (ACKD) is a condition which develops in ESRD patients characterized by cystic dilation of renal tubules and is associated with a unique type of RCC, namely, acquired cystic kidney disease-associated renal cell carcinoma (ACKD-RCC) [6]. The majority of RCCs occurring in ESRD and/or ACKD represent ACKD-RCC in addition to another unique RCC entity, clear-cell papillary renal cell carcinoma (ccpRCC). Both ACKD-RCC and ccpRCC are recognized entities in the 2016 World Health Organization classification of renal tumors [7]. Other RCCs can also occur in kidneys with ESRD, including the variety of histological types that occur in the general population without ESRD (Table 1). This review details knowledge of the clinicopathological characteristics of renal tumors associated with ESRD, with special emphasis on ACKD-RCC and ccpRCC. It also provides an update on our evolving knowledge, which will be particularly useful for pathologists and physicians who may encounter and treat patients with these tumors.

## 2. Methods

A search was conducted on the PubMed database (www.pubmed.gov, accessed 23 October 2021) to identify studies for potential inclusion in this review. The specific search terms used were “acquired cystic disease associated renal cell carcinoma” and “clear cell papillary renal cell carcinoma”, which yielded 180 and 309 potential publications, respectively. Studies from this search written in the English language were selected for inclusion by the authors based on relevance to the topics addressed in this review. Additional relevant studies were also included from the authors’ own libraries.

## 3. Etiology and Pathogenesis of RCC in ESRD

The biological environment present in kidneys with ESRD seems to promote tumor development, as evidenced by the fact that tumors of several histological types are more common in ESRD kidneys and that these tumors often occur multiply or multifocally within the same patient [10,11,12,13,14,15]. The histogenesis of tumors in ESRD is complex, with no single etiological factor identified; nonetheless, studies have shed light on a number of factors which may contribute to the pathogenesis of tumors in ESRD kidneys. The role of oxidative stress in ESRD kidneys is a possible factor, as markers of oxidative stress, including iNOS, 8-OHdG, and COX-2, assessed by immunohistochemistry were overexpressed in 42 dialysis kidneys compared to 51 kidneys with normal renal function [16]. The upregulation of antioxidant proteins may be a related factor in tumorigenesis, as these proteins were found to be upregulated in RCC arising in dialyzed kidneys versus RCC in non-dialyzed kidneys [17]. Specifically, peroxiredoxins were expressed at higher levels with longer durations of dialysis and in RCC in dialysis kidneys versus sporadic RCC. Peroxiredoxin and thioredoxin were also expressed highly in ACKD. Other actors in tumorigenesis in ESRD include hepatocyte growth factor (HGF), which, along with its receptor, c-met, was found to be upregulated in ACKD kidneys with RCC and in hyperplastic cysts in those kidneys [18]. Hypoxia-inducible factor protein 2 (HIP-2), hypoxia-inducible factor 1-alpha (HIF-1-alpha), and phosphorylated nuclear factor-kappa B (NF-kB) were also upregulated in nontumor and tumor areas in ACKD kidneys compared to non-ACKD kidneys [19]. Hyperplastic cysts in those kidneys predominantly expressed HIP-2 and HIF-1-alpha. In addition, HIP-2 was detected predominantly in papillary renal cell carcinoma, whereas HIF-1-alph and NF-kB were predominant in clear-cell renal cell carcinoma [19]. Mutations in mitochondrial DNA may also play a role, as 94 sequence variations in a variety of areas of the mitochondrial genome were detected in ESRD kidneys and tumors arising in ESRD [20].

**Figure 1:**
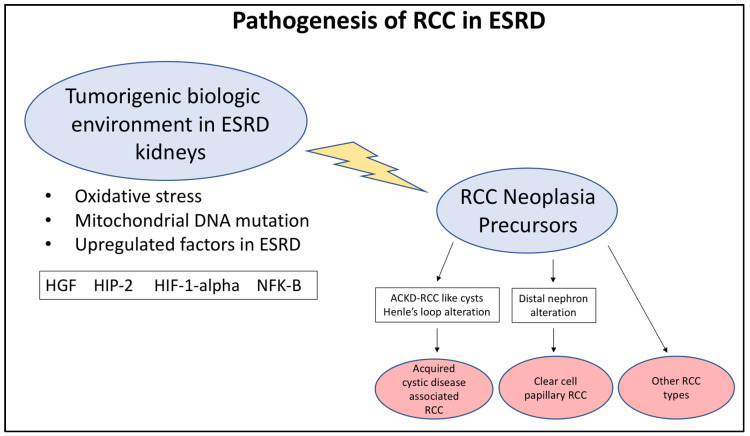
Summary of pathogenesis of RCC in ESRD.

The origins of ACKD-RCC and ccpRCC may be related to the background cysts in ACKD, which may be precursor lesions to these tumors, as proposed in a recent study which analyzed 16 cysts in ESRD kidneys deemed “ACD-RCC-like cysts” [21]. Another study classified cysts in ESRD into either clear-cell, foamy, or eosinophilic types, and found features of these cysts that suggested an association of clear-cell cysts with ccpRCC and foamy or eosinophilic cysts with ACKD-RCC and papillary RCC [22]. Paneth-like cells were also found in cases of ACKD-RCC and almost all cysts of kidneys with ACKD-RCC analyzed in another study, suggesting that Paneth-like cells in these cysts may also be related to ACKD-RCC tumor development [23]. The earliest origins of ACKD-RCC may be related to cells from Henle’s loop, as one study demonstrated expression of CD57 in ACKD-RCC, which is also expressed in Henle’s loop [24]. ccpRCC on the other hand may originate from the distal nephron, as evidenced by a high frequency of immunoreactivity for GATA-3 and high molecular weight cytokeratin [25]. Figure 1 summarizes the variety of pathogenic and etiological factors for RCC in ESRD.

## 4. Clinical Features of ACKD-RCC and ccpRCC

### 4.1. Epidemiology and Risk Factors

Various reports include data on the incidence of ACKD-RCC and ccpRCC. ccpRCC is the fourth most common subtype of RCC in the general population, representing 4.1% of all RCCs in 290 consecutive nephrectomies [26]. Although it appears that ccpRCC has a significantly higher frequency in ESRD patients than in the general non-ESRD population in the USA, the incidence of ccpRCC in ESRD in other countries has been reported as lower: 1.0% in 291 ESRD patients from Japan [27], 1.1% in 928 ESRD or non-ESRD nephrectomies in Hungary [28], and 5% in 37 ESRD patients from Brazil [29]. In the USA, ccpRCC frequency was 18% of 109 tumors from 61 ESRD patients [9], 14–21% in 66 ESRD only patients [8], and 21–23% in 61 ESRD and ACKD patients [8]. The USA data also show that ccpRCC incidence within ESRD groups are roughly equal for those with or without ACKD.

Risk factors associated with ACKD-RCC and ccpRCC are summarized in Table 2. ACKD-RCC has not been reported in the general population nor has it been reported in ESRD only patients without ACKD. ACKD is the most frequent type of RCC in ESRD and ACKD combined, accounting for 12–46% of all RCCs in this population [8,9,27,29]. Thus, ACKD-RCC risk is linked to mechanisms occurring in ESRD with ACKD. ACKD occurs more commonly in patients with longer durations of dialysis [30]. Longer duration of dialysis, along with young age and male sex, were associated with more frequent occurrence of ACKD-RCC [27]. There do not appear to be specific risk factors nor a sex predilection for ccpRCC [7,31].

### 4.2. Clinical Diagnosis, Prognosis, and Management

ACKD-RCC and ccpRCC may be clinically detected incidentally on imaging or after nephrectomy of an end-stage kidney for non-tumor-related indications. The definitive diagnosis of both tumors requires pathological examination of tissue, as radiological features between ACKD-RCC and ccpRCC may overlap with other entities, including urothelial carcinoma [32] or conventional clear-cell renal cell carcinoma (ccRCC) [33]. ccpRCC may be seen as solid or cystic lesions on computed tomography (CT) or magnetic resonance imaging (MRI) scans [33,34]. Solid-type ccpRCC showed high signal T2 intensity and early arterial enhancement, similar to conventional clear-cell renal cell carcinoma [34]. ccpRCC, also, can show heterogeneous hyperenhancement similar to conventional clear-cell renal cell carcinoma [35], and some ccpRCC tumors were found to have features of papillary renal cell carcinoma on imaging, namely, showing as solid with low-level enhancement [35]. On serial imaging, the rate of growth of ccpRCC was similar to that of other low stage renal cell carcinomas [35].

In a large recent cohort that included 112 ACKD-RCC tumors, prognosis of ACKD-RCC in terms of cancer-specific survival and recurrence-free survival were comparable with clear-cell renal cell carcinoma and better than papillary renal cell carcinoma [10]. Unfavorable factors for ACKD-RCC prognosis included longer durations of dialysis, tumor size, high pathological stage and grade, presence of lymphovasular invasion, and presence of sarcomatoid components [10].

In contrast to ACKD-RCC, ccpRCC is an indolent tumor, with the overwhelming majority of ccpRCC tumors reported in several studies demonstrating a lack of metastasis or recurrence in patients with ccpRCC [11,28,36,37,38,39,40]. One case of ccpRCC was reported with metastasis and sarcomatoid differentiation [41]. This case had exclusive ccpRCC morphology in the non-sarcomatoid areas and immunohistochemical and molecular evidence supporting a diagnosis of ccpRCC rather than sarcomatoid conventional clear-cell renal cell carcinoma. However, in the absence of sarcomatoid differentiation or other high-risk features, ccpRCC may be clinically managed as a tumor of very low potential for aggressive behavior.

In contrast to ccpRCC, management of ACKD-RCC should be less conservative. For one patient with ACKD-RCC with multiple lung and lymph node metastases refractory to nivolumab therapy, axitinib therapy led to stable disease and tumor shrinkage without adverse drug-related events [42].

## 5. Pathological Features of RCC Associated with ESRD

### 5.1. Overview of RCC Pathology in ESRD

All major types of RCC have been reported in ESRD kidneys, albeit at frequencies that are different from those occurring in the general population. ACKD-RCC occurs exclusively in ESRD kidneys with ACKD. ccpRCC can occur in kidneys with ESRD only, kidneys with ESRD and ACKD, or in non-ESRD kidneys. The frequency of ccpRCC is similar in ESRD-only kidneys and ESRD kidneys with ACKD, yet is much less frequent in the non-ESRD population. Clear-cell RCC (ccRCC) and papillary RCC (pRCC) account for a significant proportion of RCC in ESRD. The morphological types of ESRD-associated RCCs and their frequencies are summarized in Table 1.

Multifocality and bilaterality are frequent in ESRD-associated RCC and the frequency is very high for papillary RCC and ACKD-RCC, but much lower for other types of RCC [9]. More than one type of RCC can develop in a single kidney and the most frequent combination seems to be ACKD-RCC and other RCC types, especially papillary RCC [9]. The morphological features of RCC in ESRD kidneys remain similar to those in the general population. The remainder of this review will focus on the pathological features of the two most common subtypes of RCC in ESRD, namely, ACKD-RCC and ccpRCC.

### 5.2. Pathological Features of ACKD-RCC

#### 5.2.1. Macroscopic Features

**Figure 2:**
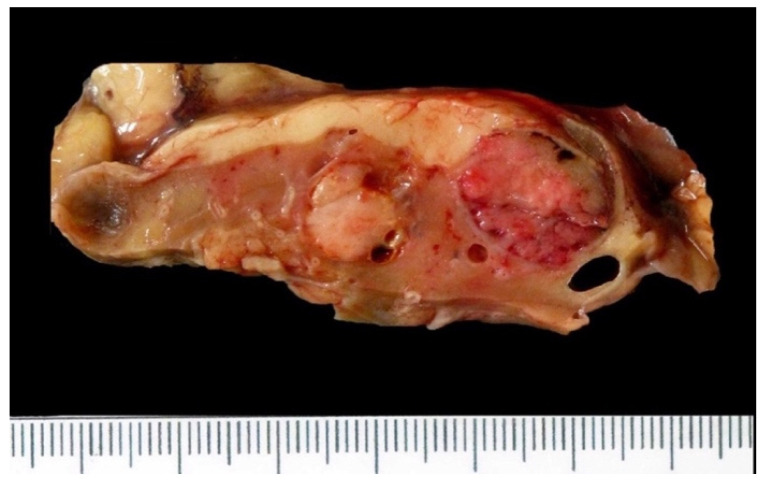
Concurrent ACKD-RCC and ccpRCC in a single kidney. Two tumors occurred simultaneously in this nephrectomy, a ccpRCC (right side of picture, abutting renal capsule) and a smaller ACKD-RCC (central part of picture).

The background kidney tissue displays changes typical of ESRD, namely, atrophic kidneys with ill-defined corticomedullary junctions. There are also multiple cysts representing acquired cystic disease [43]. Tumor masses are variable in size (0.2–8.5 cm, mean 2.7 cm), with a brown, red-brown, or yellow cut surface, often with hemorrhage and/or necrosis [44]. It may be solid, solid/cystic, or appear to arise from the background renal cysts [8,44,45]. The tumors may be multifocal, bilateral, or occur simultaneously with RCCs of other histological types (Figure 2) or benign renal tumor types [45]. Multiple tumor masses, each with typical features of ACKD-RCC, can be seen in more than a third of cases [9]. The most frequent other RCC type occurring with ACKD-RCC is papillary RCC, but other types, including clear-cell RCC or ccpRCC, may also occur [9].

In the background non-neoplastic ACKD kidney, secondary changes, including coagulative/fibrinous necrosis of cyst contents and potentially massive bleeding from cysts, are often observed and may be of an extent that simulates tumors clinically, even on imaging. Nephrectomy is often performed for these ambiguous lesions, and RCC is first detected on tissue examination [46], highlighting the need for careful gross examination of such kidneys by the pathologist.

#### 5.2.2. Microscopic Features

**Figure 3:**
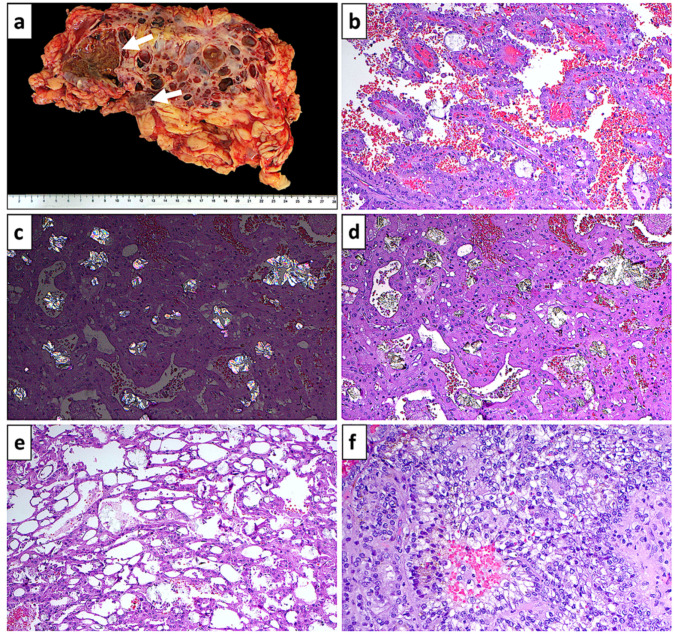
Gross and histological features of ACKD-RCC. (**a**) ACKD-RCC arising in two foci (arrows) of a kidney with multiple background cysts. (**b**) Papillary architecture in ACKD-RCC. (**c**) Calcium oxalate crystals in ACKD-RCC under polarized light. (**d**) Nests and cords of eosinophilic cells in ACKD-RCC with interspersed calcium oxalate. (**e**) Characteristic “sieve-like” spaces. (**f**) Area of an ACKD-RCC, including cells with clear cytoplasm. (Note: (**a**) gross image, (**b**–**f**) photomicrographs of hematoxylin and eosin stained sections, (**b**–**e**) 100× magnification, (**f**) 200× magnification).

ACKD-RCC shows characteristic tumor cells with abundant eosinophilic or clear cytoplasm, prominent nucleoli, and cytoplasmic or intercellular vacuolation, giving a “sieve-like appearance”. Morphological heterogeneity is frequent and includes papillary, solid, microcystic, and tubular architectures. A characteristic feature is the presence of intratumoral calcium oxalate crystals, which are often present but not absolutely necessary for the diagnosis [7]. Sarcomatoid [44,47,48] and rhabdoid [44,48] features have been described, as have other microscopic features, including degenerated foamy tumor cells, hemorrhage, intracytoplasmic hemosiderin, and psammoma bodies [45,49]. The WHO/ISUP tumor grades are more commonly 3 or 4 in ACKD-RCC as compared with conventional clear-cell RCC or papillary RCC; however, sarcomatoid features, lymphovascular invasion, and tumor necrosis are less frequent in ACKD-RCC [27]. Figure 3 displays common gross and histological features of ACKD-RCC.

#### 5.2.3. Immunophenotype

Studies of cases of ACKD-RCC with immunohistochemical staining results are summarized in Table 3. ACKD-RCC was shown to be positive for AMACR [44,45,49] and negative or patchy for cytokeratin 7 [44,45]. One study showed consistent positivity for CD10 [49]. PAX8 is expected to be positive in RCC; however, a minor subset (5 out of 24 cases) were negative in one study [45]. CD117 had varied results across two studies [45,49]. Fumarate hydratase (FH) loss, which characterizes the recently recognized entity of FH-deficient RCC, was not detected in any cases of ACKD-RCC [45]. One case of ACKD-RCC with sarcomatoid features showed diffuse p53 positivity in the sarcomatoid component [47]. Other positive markers included napsin A [50], pan cytokerain, PTEN, and C-Met [49], and negative markers included CAIX, CD57, CD68, PDGFR, PAX-2, and VEGFR-2 [49]. Kidney-specific cadherin showed heterogenous staining [49].

#### 5.2.4. Molecular Characteristics

Most studies have assessed molecular alterations in ACKD-RCC at the chromosomal level [47,48,51,52,53,54,55]. The detected alterations vary between studies and even between tumors in the same patient [53], showing a lack of constant or specific chromosomal alterations in ACKD-RCC. This finding contrasts with those regarding several recently recognized entities of RCC which are defined by specific molecular alterations. Of note, these molecular profiles are tumor type-specific but distinctly different from the profiles of other well recognized RCC types, such as conventional clear-cell RCC (chromosome 3p deletions) and papillary RCC (gains of chromosomes 7 and 17 and deletions of Y chromosomes) [7]. Interestingly, one study based on comparative genomic hybridization analysis [56] showed that ACKD-RCC, ccpRCC, and papillary RCC occurring in ESRD kidneys clustered together in a group distinct from ccRCC. At the genetic level, one recent study suggests that recurrent mutations in KMT2C and TSC2 genes occur in ACKD-RCC [57]. These mutations occurred in four out of five and three out of five ACKD-RCC cases, respectively. Additional mutations in CBL, PDGFRA, and SYNE1 genes were detected but were non-recurrent and coexisted with KMT2C and TSC2 mutations. Loss of expression of BAP-1 protein, which has been found in a subset of aggressive RCC cases [58], was not detected in four cases of ACKD-RCC [59].

Thus, overall ACKD-RCC lacks specific or defining molecular alteration, yet the available number of studies and the number of tumors analyzed at the molecular level are relatively small. More investigation is likely needed to fully characterize the molecular profile of ACKD-RCC.

### 5.3. Pathological Features of ccpRCC

#### 5.3.1. Macroscopic Features

In a series of 55 ccpRCC tumors [39], size ranged from 0.2–7.5 cm and they were variably cystic or solid, with white-tan, pale yellow, or red-brown congested cut surfaces. The cystic components of tumors included multilocular cysts with solid nodules within cystic septa or at the junction of the cysts with renal parenchyma. Almost all of the tumors had at least some cystic component. When predominantly solid, the cysts were arranged at the periphery of solid areas with angulated, flattened, or irregular contours of cysts. All of the tumors were at least partially pseudo-encapsulated or completely encapsulated [15,39]. The bright yellow, fleshy, or heterogenous cut surfaces, typically seen in conventional ccRCC, were not present—an important differential diagnostic clue. Necrosis, venous invasion, or renal sinus invasion was not seen. Calcification or ossification may be observed.

#### 5.3.2. Microscopic Features

**Figure 4:**
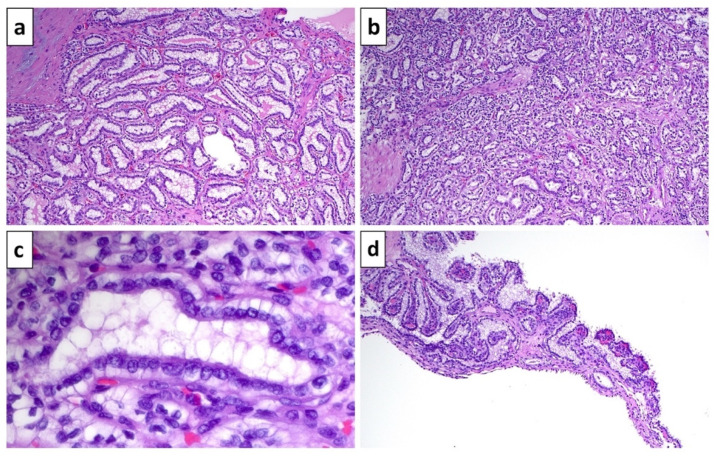
Histological features of ccpRCC. (**a**) Tubular architecture in ccpRCC. (**b**) Areas with tubular and solid or nested architecture of clear cells. (**c**) Clear cells with linear arrangement of nuclei and inverse polarization, which have been described as resembling secretory endometrial glands. (**d**) Cystic septum in ccpRCC with short papillary projections. (Note: Hematoxylin and eosin stained sections, (**a**,**b**,**d**) 100× magnification, (**c**) 600× magnification).

Histologically, ccpRCC is characterized by cuboidal to columnar clear cells with a tubulo-papillary arrangement. Virtually all tumors show at least focal papillary architecture [39]. Microcystic or acinar architecture can also be present [15]. Foamy macrophages or psammoma bodies, characteristic for papillary RCC, are not seen [39]. Some tumors show a garland arrangement around hyalinized or fibrous zones [39]. One of the most characteristic findings is a linear arrangement of tumor nuclei, lining up with inverted polarity away from the basement membrane. Low-grade nuclei are noted in the majority of cases, with WHO/ISUP grade 2 nuclei being the most common, and a small subset showing grades 1 or 3 [39]. Tumor necrosis, rhabdoid or sarcomatoid features, or giant cells are highly unusual, and their presence should call for a consideration of other RCC types [28]. Figure 4 displays common gross and histological features of ccpRCC.

Ultrastructurally, ccpRCC displayed short microvilli, cytoplasmic interdigitations, nuclear pseudoinclusions, and stromal myofibroblasts [40].

#### 5.3.3. Immunophenotype

Studies with results of immunohistochemical staining for cases of ccpRCC are summarized in Table 4. It appears, according to the available literature, that staining patterns, whether in ESRD or non-ESRD kidneys, are essentially identical.

Cytokeratin 7 [14,15,39,60,61,62,63] and CAIX [39,60,61,63] were consistently positive in cases of ccpRCC across studies. High-molecular weight cytokeratin (HMWCK) [64] and vimentin were each found to be positive in respective series [15]. AMACR is weak or negative in the majority of ccpRCC cases [14,15,39,60,61,63]—a feature which is helpful in differentiating ccpRCC from papillary RCC. As expected in a tumor of renal origin, PAX-8 is positive [63]. RCC antigen was negative in a majority of cases studied [62], a finding which may be useful in distinguishing ccprCC from conventional clear-cell RCC. Immunostaining for CD-10 is not reliable in ccpRCC, with various staining results across tumors and studies [14,15,39,60,61]. GATA3 positivity was seen in the majority of cases of ccpRCC, while it was negative in conventional ccRCC and pRCC [65]. GATA3 positivity is a finding which ought to be taken into consideration should urothelial carcinoma, which is typically also GATA3-positive, enter the differential diagnosis. A variety of other markers which showed positivity in individual series included napsin A [50], cyclin D1 [66], parafibromin [62], and vitamin D receptor [67]. TFE3, which is a positive marker for Xp11 translocation RCC, was negative across three studies [15,60,61]. Estrogen and progesterone receptor staining was negative in one study [39].

#### 5.3.4. Molecular Characteristics

Compared to conventional clear-cell RCC, ccpRCC showed a low mutational burden [68] and fewer somatic mutations [36]. In a study of 18 patients with ccpRCC compared with the Cancer Genome Atlas cohort of ccRCC and papillary RCC, the overall mutational characteristics of ccpRCC were distinct [69].

Although earlier studies showed no mutations in the Von Hippel Lindau (VHL) gene in sets of ccpRCC cases [14,61,70,71], recent studies of ccpRCC have shown that some cases may harbor mutations in VHL, an alteration typically associated with ccRCC [69,72]. ccpRCC tumors have been seen in patients with germline VHL syndrome [73,74], suggesting that alterations in VHL may play a role in the development of ccpRCC, as they do in ccRCC. A set of “clear-cell papillary-like tumors” with morphological features typical of ccpRCC but immunohistochemical staining not typical of ccpRCC has also been described in patients with VHL disease [39]. A case from TCGA was noted in one study to show promotor hypermethylation of VHL [75]. However, there is debate as to whether cases of ccpRCC with VHL alterations represent true ccpRCC, and it has been suggested that such tumors may actually be conventional ccRCC misclassified as ccpRCC [25].

Overall, there is a lack of recurrent molecular alterations in all cases of ccpRCC. However, studies of ccpRCC have detected specific molecular alterations in subsets of cases of this tumor. Out of 18 ccpRCC tumors, 3 showed TCEB-1 mutations, and 8 out of 18 ccpRCC tumors showed increased frequency of germline variants associated with Fanconi anemia [69]. Out of 14 ccpRCC tumors, 3 showed mutations in the MET protooncogene [76]. BAP-1 loss, a finding associated with more aggressive behavior of a subset of ccRCC tumors, was detected in 1 out of 4 cases of ccpRCC [59]. However, no germline BAP-1 mutations were detected in 18 cases in another study [69]. Germline alterations in fumarate hydratase and MET were also absent [69]. Interestingly, alterations in metabolism were prominent findings in cases of ccpRCC in some series [68]. These alterations included depletion of mitochondrial DNA [36,68], decreased mitochondrial RNA expression, and increased levels of sorbitol, glutathione, and NADH [68]. Chromosomal alterations in chromosomes 3, 6, 7, 12, 16, 17, and 20 have been noted in studies [14,60,64,72,74], although none of these alterations was recurrent across all cases of ccpRCC.

Micro-RNA (miRNA) expression has been assessed in some studies [76,77,78]. A study of 15 cases of ccpRCC found that miRNA expression patterns differed from normal renal parenchyma [77]. The same study found that miRNA expression in ccpRCC had some overlap with that of ccRCC and pRCC; however, ccpRCC lacked the dysregulation of miRNAs that has been associated with aggressive behavior [77]. Another study found that members of the miRNA-200 family were overexpressed in ccpRCC [76]. Expressions of other RNA types analyzed in that study were also different from cases of ccRCC and pRCC [76]. A case report of one case of ccpRCC in an ESRD kidney showed that miRNA-155 was upregulated in tumor tissue compared to non-tumor renal cortex, and expression of miRNA-155 was greater in ESRD renal cortex than in non-dialyzed kidney tissue [78].

Overall, the molecular profile of ccpRCC appears to be different from other recognized RCC entities, supporting its classification as a unique entity. Also based on available molecular data, the pathogenesis of ccpRCC appears to rely on a variety of molecular mechanisms; however, it is as yet not certain which, if any, of these molecular mechanisms is the ultimate driver of ccpRCC tumor development. It is also as yet not clear whether ccpRCC occurring in ESRD differs at the molecular level from ccpRCC occurring in the non-ESRD population, and this may be an interesting avenue for future study.

## 6. Differential Diagnoses

Other renal cell tumors may enter the differential diagnosis of ACKD-RCC and ccpRCC, and accurate diagnosis is important for prognostication and clinical treatment decisions. Table 5 summarizes the most important diagnostic features of ACKD-RCC and ccpRCC and other RCC types in the differential diagnosis.

ACKD-RCC may resemble other tumors which do occur in ESRD, including papillary RCC, chromophobe RCC, or other less frequent subtypes of RCC, such as MiT translocation RCC and fumarate hydratase-deficient RCC. The prerequisite for the presence of ACKD for the diagnosis of ACKD-RCC and distinctive histological features of cribriform architecture and the presence of intratumoral calcium oxalate are the most helpful in making the correct diagnosis. Immunohistochemistry may also help rule out other types of RCC; however, ACKD-RCC does not show a diagnostically consistent or specific immunophenotype.

The main histological differential diagnosis of ccpRCC is conventional clear-cell RCC. Increasing the diagnostic difficulty is the fact that many clear-cell tumors harbor overlapping histological and immunohistochemical features between ccpRCC and ccRCC. Studies of tumors with overlapping features [79,80] showed that such tumors may behave more aggressively compared to tumors with pure ccpRCC morphology. Thus, care must be taken for appropriate gross sampling and in the diagnosis of such tumors from limited biopsy samples. Of note, one recent study showed that attention to intratumoral vascular patterns may help distinguish ccRCC from ccpRCC, as lacuar and pseudoacinar patterns were more common in ccRCC, whereas Golgi-like in addition to lacunar patterns were more frequent in ccpRCC [81]. Papillary renal cell carcinoma with clear cytoplasm may also enter the differential diagnosis of ccpRCC. The presence of foamy macrophages or psammoma bodies, clear vacuolated rather than clear-empty cytoplasm, and positivity for AMACR and negativity for CAIX immunostains are features of pRCC which help make the distinction from ccpRCC [25]. Cystic ccpRCC may also resemble multilocular renal neoplasm of low malignant potential (MCCN-LMP), as one study showed 5 out of 9 cases previously diagnosed as MCCN-LMP were actually ccpRCC [82]. A case of Xp11 translocation RCC diagnosed by TFE3 immunohistochemistry and showing aggressive clinical behavior was also reported to show morphological overlap with ccpRCC [83].

## 7. Conclusions

Knowledge of the unique characteristics of RCC in ESRD continues to evolve. It is likely that pathologists and other physicians treating patients with ESRD will encounter these tumors, particularly ACKD-RCC or ccpRCC, which are the most frequent in this setting. Knowledge of the biology of these tumors is essential for clinicians who will make treatment decisions for RCC in ESRD. This is especially true in light of the increasing numbers of ESRD patients worldwide. Future studies may further address the precise etiological factors, molecular pathology, and potential immunophenotypic markers which could aid in diagnosing and treating patients with these tumors.

## Figures and Tables

**Table 1:** Major RCC types and frequencies in ESRD, ACKD, and among all renal cell tumors.

	ESRD Only (*n* = 36) ^1^	ESRD and ACKD (*n* = 91) ^1^	All Renal Cell Tumors (WHO 2016)
ACKD-RCC	0%	40%	Not applicable
ccpRCC	25%	30%	1–4%
Clear-cell RCC	22%	14%	65–70%
Papillary RCC	21%	17%	18.5%
Chromophobe RCC	14%	6%	5–7%

^1^ Percentages derived from data of the studies of Tickoo et al. [8] and Bhatnagar et al. [9]. Percentages for each tumor type are calculated as number of tumors per total number of ESRD kidneys. (As some tumors occurred multiply in the same kidney, the sum of percentages calculated is not 100%).

**Table 2:** Risk factors associated with ACKD-RCC and ccpRCC.

**ACKD-RCC**	Acquired Cystic Renal Disease (Prerequisite Factor) Long Duration of Dialysis Young Patient Age Male Sex
**ccpRCC**	No known specific risk factors

**Table 3:** Immunohistochemical markers in ACKD-RCC *.

Marker	Number of Positive Cases *n* (Study)	Total Positive Cases *n* (%)	Number of Negative Cases *n* (Study)	Total Negative Cases *n* (%)	Other Staining Patterns
AMACR	24 (Przybycin 2018)		0 (Przybycin 2018)		-
	12 (Ahn 2013)		0 (Ahn 2013)		
	5 (Kuroda 2017)		0 (Kuroda 2017)		
	6 (Kuroda 2011)	47 (100%)	0 (Kuroda 2011)	0 (0%)	
Cytokeratin 7	0 (Kuroda 2017)		5 (Kuroda 2017)		20 negative to patchy (Przbycin 2018)
			2 (Kuroda 2011)		
	1 (Kuroda 2011)				3 cases focally positive (Kuroda 2011)
		1 (13%)		7 (87%)	
PAX8	19 (Przybycin 2018)	19 (79%)	5 (Przybycin 2018)	5 (21%)	-
CD117	24 (Przybycin 2018)		0 (Przybycin 2018)		-
	0 (Ahn 2013)	24 (67%)	12 (Ahn 2013)	12 (33%)	
CD10	12 (Ahn 2013)	12 (100%)	0 (Ahn 2013)	0 (0%)	-
Napsin A	-	-	-	-	2 cases with cytoplasmic dot positivity (Zhu 2015)
Fumarate hydratase	-	-	-	-	24 retained (Przybycin 2018)
P53	-	-	-	-	1 diffuse in sarcomatoid component (Tajima 2015)
Pan cytokeratin	12 (Ahn 2013)	12 (100%)	0 (Ahn 2013)	0 (0%)	-
PTEN	12 (Ahn 2013)	12 (100%)	0 (Ahn 2013)	0 (0%)	-
C-Met	12 (Ahn 2013)	12 (100%)	0 (Ahn 2013)	0 (0%)	-
CA-IX	0 (Ahn 2013)	0 (0%)	12 (Ahn 2013)	12 (100%)	-
CD57	0 (Ahn 2013)	0 (0%)	12 (Ahn 2013)	12 (100%)	
CD68	0 (Ahn 2013)	0 (0%)	12 (Ahn 2013)	12 (100%)	-
PDGFR	0 (Ahn 2013)	0 (0%)	12 (Ahn 2013)	12 (100%)	-
PAX-2	0 (Ahn 2013)	0 (0%)	12 (Ahn 2013)	12 (100%)	3 cases focally positive (Kuroda 2011)
	2 (Kuroda 2011)	2 (13%)	1 (Kuroda 2010)	13 (87%)	
VEGFR-2	0 (Ahn 2013)	0 (0%)	12 (Ahn 2013)	12 (100%)	-
Kidney-Specific Cadherin	-	-	-	-	12 heterogeneous (Ahn 2013)
Antimitoch-ondrial Antibody	6 (Kuroda 2011)	6 (100%)	0 (Kuroda 2011)	0 (0%)	

* Please see Table A1 in Appendix A for more detailed information about study sizes, populations, and tumor histologies in the cited studies.

**Table 4:** Immunohistochemical markers in ccpRCC *.

Marker	Number of Positive Cases *n* (Study)	Total Positive Cases *n* (%)	Number of Negative Cases *n* (Study)	Total Negative Cases *n* (%)	Other Staining Patterns
Cytokerain 7	34 (Williamson 2013)		0 (Williamson 2013)		-
	7 (Gobbo 2008)		0 (Gobbo 2008)		
	1 (Kuroda 2011)		0 (Kuroda 2011)		
	9 (Rohan 2011)		0 (Rohan 2011)		
	20 (Pramick 2013)		0 (Pramick 2013)		
	20 ** (Cui 2013)		0 (Cui 2013)		
	15 *** (Park 2012)	106 (100%)	0 (Park 2012)	0 (0%)	
CAIX	34 (Williamson 2013)		0 (Williamson 2013)		-
	7 (Gobbo 2008)		0 (Gobbo 2008)		
	9 (Rohan 2011)		0 (Rohan 2011)		
	18 (Pramick 2013)	68 (100%)	0 (Pramick 2013)	0 (0%)	
AMACR	1 **** (Williamson 2013)		0 (Williamson 2013)		-
	0 (Gobbo 2008)		7 (Gobbo 2008)		
	0 (Kuroda 2011)		1 (Kuroda 2011)		
	0 (Rohan 2011)		9 (Rohan 2011)		
	0 (Park 2012)		15 (Park 2012)		
	1 (Pramick 2013)	2 (4%)	19 (Pramick 2013)	51 (96%)	
CD10	0 (Gobbo 2008)		7 (Gobbo 2008)		20 of 34 cases, patchy luminal membranous staining of cystic components only (Williamson 2013)
	0 (Kuroda 2011)		1 (Kuroda 2011)		
	0 (Rohan 2011)		9 (Rohan 2011)		
	0 (Park 2012)	0 (0%)	15 ***** (Park 2012)	32 (100%)	
PAX8	20 (Pramick 2013)	20 (100%)	0 (Pramick 2013)	0 (0%)	-
HMWCK	12 (Martignoni 2017)		1 (Martignoni 2017)		-
	1 (Gilani 2012)	13 (93%)	0 (Gilani 2012)	7 (7%)	
GATA3	19 (Mantilla 2017)	19 (76%)	6 (Mantilla 2017)	(24%)	-
RCC	0 (Cui 2013)	0 (0%)	20 * (Cui 2013)	20 (100%)	-
TFE3	0 (Gobbo 2008)		7 (Gobbo 2008)		-
	0 (Rohan 2011)		9 (Rohan 2011)		
	0 (Park 2012)	0 (0%)	15 (Park 2012)	31 (100%)	
Vimentin	15 (Park 2012)	15 (100%)	0 (Park 2012)	0 (0%)	-
Napsin A	-	-	-	-	9 out of 19 cases showed cytoplasmic dot positivity (Zhu 2015)
Cyclin D1	35 (Leroy 2014)	35 (100%)	0 (Leroy 2014)	0 (0%)	7 cases with focal staining (Leroy 2014)
Parafibromin	20 (Cui 2013)	20 (100%)	0 (Cui 2013)	0 (0%)	-
Estrogen Receptor	0 (Williamson 2013)	0 (0%)	29 (Williamson 2013)	29 (100%)	-
Progesterone Receptor	0 (Williamson 2013)	0 (0%)	29 (Williamson 2013)	29 (100%)	-
Vitamin D Receptor	21 (Wang 2018)	21 (100%)	0 (Wang 2018)	0 (Wang 2018)	5 cases intermediate (Wang 2018)

* Please see Table A1 in Appendix A for more detailed information about the study sizes, populations, and tumor histologies studied. ** A majority but not all of the 20 cases were positive. *** Moderate to intense staining. **** Weak, granular cytoplasmic staining. ***** Negative or focal weak staining.

**Table 5:** Summary of diagnostic features and differential diagnosis of ACKD-RCC and ccpRCC.

	ACKD-RCC	ccpRCC
Background Kidney	Presence of ACKD in background ESRD kidney	Occurs with or without ACKD or ESRD
Tumor Histology	“Sieve-like morphology”, calcium oxalate crystals, frequently has high nuclear grade (WHO/ISUP grade 3 or 4)	Tubulopapillary architecture, linearly arranged nuclei with inverted polarity, majority of cases have low nuclear grade (WHO/ISUP 1 or 2)
Immunohistochemistry	CK7−, AMACR+	CK7+, AMACR−, HMWCK+, GATA3+
Molecular	No one specific molecular marker	No one specific molecular marker
Differential Diagnosis	Papillary RCC Chromophobe RCC MiT translocation RCC FH-deficient RCC	Clear-cell (conventional) RCC Papillary RCC with cytoplasmic clearing Multilocular renal neoplasm of low malignant potential MiT transloation RCC

## Data Availability

Not applicable.

## Appendix A

**Table A1:** Supplementary information from studies of immunohistochemistry in ESRD-associated RCC.

Study	Total Study Size	Tumors Assessed by IHC	Patient Population	Tumor Histologies
Przybycin 2018	40 tumors	24 tumors	32 male, 8 female 24–79 years (mean 52 years)	ACKD-RCC (all tumors)
Ahn 2013	12 tumors	12 tumors	10 male, 2 female 35–70 years (mean 48.7 years)	ccpRCC (all tumors)
Kuroda 2017	7 tumor	7 tumors	5 male, 2 female 38–78 years (mean 52.9 years)	ACKD-RCC (all tumors)
Zhu 2015	159 tumors	159 tumors	Not documented	ACKD-RCC (2 tumors), ccpRCC (19 tumors), other RCC types (138 tumors)
Tajima 2015	1 tumor	1 tumor	77-year-old male	ACKD-RCC with sarcomatoid component
Williamson 2013	55 tumors	34 tumors	19 male, 15 female 33–87 years (mean 61 years)	ccpRCC (all tumors)
Gobbo 2008	7 tumors	7 tumors	3 male, 2 female 53–64 years (mean 60 years)	ccpRCC (all tumors)
Kuroda 2011	1 tumor	1 tumor	57-year-old male	ccpRCC
Kuroda 2011	6 tumors	6 tumors	6 male, 0 female 44–74 years (mean 59.7 years)	ACKD-RCC, including 1 with sarcomatoid change
Rohan 2011	9 tumors	9 tumors	5 male, 4 female 46–74 years (mean 61.7 years)	ccpRCC (all tumors)
Pramick 2013	20 tumors	20 tumors	12 male, 8 female 27–76 years. (mean 59 years)	ccpRCC (all tumors)
Cui 2013	20 tumors	20 tumors	11 male, 9 female 23–70 years. (mean 55 years.)	ccpRCC (all tumors)
Park 2012	15 tumors	15 tumors	4 male, 11 female 35–70 years (mean 52 years)	ccpRCC (all tumors)
Martignoni 2017	14 tumors	14 tumors	9 male, 5 female 46–77 years (mean 61 years)	ccpRCC (all tumors)
Gilani 2012	1 tumor	1 tumor	70 year old female	ccpRCC
Mantilla 2017	210 tumors	210 tumors	3:1 male/femle ratio, mean age 64 years *	ccpRCC (25 tumors), ccRCC (109 tumors), papillary RCC (62 tumors), others (14 tumors)
Leroy 2014	42 tumors	42 tumors	25 male, 17 female 35–78 years (mean 60.7 years)	ccpRCC (all tumors)
Wang 2018	26 tumors	26 tumors	19 male, 7 female 36–74 years (mean 53.5 years)	ccpRCC (all tumors)

* These data are for the 25 ccpRCC patients only.
